# *Helicobacter pylori* In Free‐Living Amoebae: A Potential Environmental Reservoir for Persistence and Transmission

**DOI:** 10.1111/1758-2229.70436

**Published:** 2026-10-16

**Authors:** Otmane Lamrabet, Meriem Bekliz, Shamsul Qumar, Merylin Sebastian, Ula Abdurahman, Mohamad Hamad, Lucy Semerjian, Ghalia Khoder

**Affiliations:** ^1^ Department of Applied Biology, College of Sciences University of Sharjah, University City Sharjah UAE; ^2^ Faculty of Medicine University of Geneva Geneva Switzerland; ^3^ Research Institute for Medical & Health Sciences, University of Sharjah Sharjah UAE; ^4^ Department of Medical Laboratory Sciences, College of Health Sciences University of Sharjah Sharjah UAE; ^5^ Department of Environmental Health Sciences, College of Health Sciences University of Sharjah Sharjah UAE; ^6^ Water and Environment Conservation Research Center, Research Institute of Science and Engineering, University of Sharjah Sharjah UAE; ^7^ Department of Pharmaceutics and Pharmaceuticals Technology, College of Pharmacy University of Sharjah Sharjah UAE

**Keywords:** amoeba‐resistant bacteria, coccoid form, environmental reservoirs, free‐living amoebae, *Helicobacter pylori*, persistence, viable but non‐culturable form, waterborne transmission

## Abstract

*Helicobacter pylori* ( *H. pylori* ) is a highly adapted gastric pathogen responsible for one of the most prevalent chronic bacterial infections worldwide and a major cause of gastritis, peptic‐ulcer disease, and gastric cancer. Traditionally considered predominantly a human‐associated microorganism transmitted via oral‐faecal routes, growing evidence challenges this paradigm by demonstrating its persistence outside the human host through interactions with free‐living amoebae (FLA). This review critically examines current knowledge of the biological and ecological interplay between *H. pylori* and FLA, focusing on amoeba‐mediated internalisation, intracellular survival, morphological plasticity, and protection from environmental stressors. We highlight FLA's ability to support the persistence of spiral and viable but non‐culturable (VBNC) coccoid forms of *H. pylori* , potentially enabling prolonged survival in aquatic and food‐associated environments and raising concerns regarding potential waterborne and foodborne transmission. Given the limited direct experimental evidence, we integrate insights from amoeba‐resistant bacteria to illustrate convergent intracellular persistence strategies. By considering FLA as a potential underrecognised environmental reservoir, this review expands current frameworks of *H. pylori* transmission and persistence, with potential relevance to settings with inadequate sanitation. We also outline key mechanistic and methodological gaps and call for standardised models to clarify the ecological significance of these interactions and inform surveillance and control strategies.

## Graphical abstract

Free‐living amoebae may provide an underrecognised environmental niche for *Helicobacter pylori* , supporting its intracellular persistence, morphological plasticity, and protection from environmental stress. These interactions could facilitate prolonged survival in aquatic and food‐associated environments and contribute to potential waterborne or foodborne transmission.

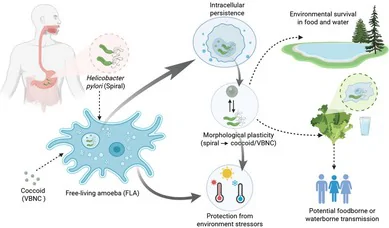

## 1 Introduction

*Helicobacter pylori* causes one of the most prevalent chronic bacterial infections worldwide, colonising approximately 4.4–4.5 billion individuals and exhibiting marked geographic heterogeneity closely linked to sanitation, hygiene, and socioeconomic conditions (Hooi et al. 2017; Khoder et al. 2019). Infection is typically acquired in early childhood and can persist asymptomatically for decades through stable colonisation of the gastric mucosa. Transmission has traditionally been attributed to direct human‐to‐human contact via oral‐oral and faecal‐oral routes, consistent with detection of *H. pylori* in saliva, vomitus, gastric refluxate, and faeces (Ferguson et al. 1993; Leung et al. 1999; Parsonnet et al. 1999). Socioeconomic and environmental conditions, including household crowding, hygiene practices, and water‐related exposures, are associated with *H. pylori* infection risk, while age‐related prevalence patterns reflect early acquisition and lifelong persistence (Salama et al. 2013; Che et al. 2023; Chen et al. 2024). Despite declining prevalence in many developed countries, *H. pylori* remains a major global health burden due to strain‐specific virulence, host susceptibility, and the rising prevalence of antimicrobial resistance, which increasingly compromises eradication efforts (Elbehiry et al. 2024; Salahi‐Niri et al. 2024; Lai et al. 2025; Shmakova et al. 2025). The recognition of clarithromycin‐resistant *H. pylori* as a priority antimicrobial‐resistant pathogen by the World Health Organization underscores the need to reassess its epidemiology and persistence beyond the human host (Dang and Graham 2017; Willyard 2017).

Free‐living amoebae (FLA), including *Acanthamoeba* spp., *Naegleria* spp., and *Dictyostelium discoideum*, are ubiquitous protozoa in aquatic and terrestrial environments, where many taxa can persist through cyst formation and association with biofilms (Fan et al. 2024). While only a limited number of FLAs are direct human pathogens, their ecological role as predators of bacteria exerts strong selective pressure favoring the emergence of amoeba‐resistant bacteria (ARB) (Balczun and Scheid 2017). Such bacteria, including *Legionella* and *Mycobacterium*, survive and replicate intracellularly within amoebae, allowing FLAs to function as environmental reservoirs and ‘Trojan horses’ that protect pathogens from environmental stressors and disinfection processes (Greub and Raoult 2004; Thomas et al. 2010; Lamrabet et al. 2012).

Emerging evidence indicates that *H. pylori* can similarly associate with FLAs, particularly *Acanthamoeba castellanii* , where co‐culture supports bacterial survival for extended periods and enables intracellular persistence within amoebal phagosomes (Winiecka‐Krusnell et al. 2002; Hojo et al. 2020). These interactions suggest that FLAs may provide a protected niche for *H. pylori* outside the human host, facilitating survival in aquatic, soil, and engineered environments (Barker et al. 1999; Moreno‐Mesonero et al. 2017, 2020; Dey et al. 2020; Soler et al. 2023). Notably, amoebal association may support persistence of viable but non‐culturable (VBNC) *H. pylori* states, allowing evasion of environmental stressors and potentially enabling reversion to infective forms under favourable conditions (Dey et al. 2020). Elucidating the interplay between *H. pylori* and FLAs expands current paradigms of transmission by implicating environmental reservoirs as potentially overlooked contributors to *H. pylori* transmission, particularly in regions with inadequate sanitation and limited access to clean water. Understanding the molecular and cellular mechanisms underlying amoeba‐mediated persistence may reveal novel survival strategies, stress‐adaptation mechanisms, and potential changes in bacterial phenotypes relevant to virulence, thereby offering insights into environmental persistence and transmission and informing more effective surveillance and intervention strategies.

This review critically synthesises current evidence on interactions between *H. pylori* and FLA, with particular emphasis on amoeba‐mediated persistence, intracellular survival, and potential for environmental transmission. We examine the mechanisms underlying *H. pylori* internalisation, survival, and morphological adaptation within amoebal hosts, including the role of viable but non‐culturable states, and compare these strategies with those described for established amoeba‐resistant bacteria. Furthermore, this review highlights key experimental limitations and knowledge gaps in the field, emphasising the need for standardised in vitro and environmentally relevant models to better define the ecological relevance of these interactions. Beyond summarising the current literature, this review highlights the broader ecological role of FLA as microbial predators, environmental reservoirs, and mediators of microorganism‐microorganism interactions that influence bacterial persistence, adaptation, and transmission in natural and engineered environments. By integrating clinical, microbiological, and environmental perspectives, we propose a conceptual framework that extends the current view of *H. pylori* beyond a strictly human‐associated pathogen to one capable of interacting with environmental microbial reservoirs. This perspective advances our understanding of microbial ecology while highlighting the importance of incorporating protozoan hosts into environmental surveillance, microbial risk assessment, and water safety strategies aimed at reducing *H. pylori* transmission and disease burden.

## 2 Biological Adaptations of *H. pylori* to Environmental Persistence

### 2.1 Structural and Physiological Plasticity Supporting Persistence

*H. pylori* is a Gram‐negative, microaerophilic, spiral‐shaped bacterium whose morphology and physiology confer remarkable adaptability to hostile environments. The spiral shape facilitates motility through viscous matrices and enhances survival in structured niches such as mucus, biofilms, and intracellular compartments (Celli et al. 2009; Martínez et al. 2016). Under nutrient limitation or environmental stress, *H. pylori* undergoes a reversible transition from its spiral form to spherical coccoid cells, frequently associated with a viable but non‐culturable (VBNC) state that enhances tolerance to acidity, oxidative stress, and antimicrobial exposure (Pinto et al. 2015; Loke et al. 2016; Ierardi et al. 2020; Sadeghloo et al. 2026). The *H. pylori* cell envelope contributes to persistence through structural modification of lipopolysaccharides (LPS), including Lewis antigen mimicry, which reduces immune recognition and inflammatory activation (Edwards et al. 2000; Sijmons et al. 2022). Peptidoglycan remodelling contributes to cell‐envelope integrity and resistance to environmental stress (Sycuro et al. 2010, 2012). *H. pylori* also releases outer membrane vesicles (OMVs), enabling delivery of enzymes, adhesins, and virulence‐associated factors that modulate surrounding microenvironments and enhance stress resistance (Mullaney et al. 2009; Olofsson et al. 2010; Turner et al. 2018; Johnston et al. 2024). Physiologically, *H. pylori* thrives under low‐oxygen and elevated carbon dioxide conditions and possesses multiple oxidative‐stress defence mechanisms, including catalase and other antioxidant systems, that help protect the bacterium from reactive oxygen species generated during host interaction or environmental stress (Wang and Maier 2004; Bury‐Moné et al. 2006). Urease remains a central fitness determinant, buffering acidic microenvironments through ammonia production and supporting survival in both gastric and extra‐gastric acidic niches (Ha et al. 2001; Bury‐Moné et al. 2006).

### 2.2 Virulence Factors as Determinants of Persistence Beyond Pathogenesis

Beyond their established roles in disease, *H. pylori* virulence factors may also contribute to environmental resilience and intracellular survival. CagA, delivered via the *cag* pathogenicity island (*cag*PAI)–encoded Type IV secretion system (T4SS), disrupts host signalling pathways and cellular homeostasis following phosphorylation of its Glu‐Pro‐Ile‐Tyr‐Ala (EPIYA) motifs by host Src‐family and Abl kinases. Phosphorylated CagA interacts with several host signalling proteins, including SHP2, Csk, and Crk, thereby altering receptor‐associated signalling and downstream kinase cascades such as the Ras–Raf–MEK–ERK/MAPK pathway. These signalling events affect cytoskeletal organisation, cell adhesion, polarity, proliferation, and inflammatory responses (Higashi, Tsutsumi, Muto, et al. 2002; Stein et al. 2002; Hatakeyama 2014, 2017). *H. pylori* infection also modulates receptor tyrosine kinase signalling, including c‐Met‐ and epidermal growth factor receptor (EGFR)‐associated pathways, which converge with downstream kinase cascades such as ERK/MAPK and contribute to alterations in epithelial‐cell adhesion, polarity, proliferation, motility, and inflammatory responses (Backert and Tegtmeyer 2017; Tegtmeyer et al. 2017).

In parallel with CagA‐mediated signalling, the *cag* T4SS contributes to activation of a distinct innate immune pathway involving bacterial ADP‐heptose. This metabolite is sensed by the cytosolic receptor alpha‐protein kinase 1 (ALPK1), which phosphorylates TRAF‐interacting protein with forkhead‐associated domain (TIFA), promoting TIFA oligomerisation and recruitment of TNF receptor‐associated factor 6 (TRAF6). Subsequent activation of the IκB kinase (IKK)‐nuclear factor kappa B (NF‐κB) pathway induces pro‐inflammatory mediators, particularly interleukin‐8 (IL‐8), thereby promoting chemotaxis and recruitment of inflammatory cells. Importantly, the ALPK1‐TIFA pathway is mechanistically distinct from direct CagA signalling, although both are associated with the *cag* T4SS‐dependent host response (Gall et al. 2017; Zhou et al. 2018; Pfannkuch et al. 2019).

Strain‐specific variation in CagA structure, particularly in the number and composition of EPIYA motifs, modulates its phosphorylation, SHP‐2 binding, and downstream cellular effects (Higashi, Tsutsumi, Fujita, et al. 2002; Naito et al. 2006). VacA enhances bacterial survival by inducing vacuolation, mitochondrial dysfunction, and immune suppression, while polymorphic variants display differential stress tolerance and cytotoxicity (Atherton et al. 1995; Cover and Blanke 2005; Yamaoka 2010). Adhesins and outer membrane proteins, including BabA, SabA, HopQ, OipA, and AlpAB, mediate attachment to host and non‐host surfaces, promoting biofilm formation and stable association with biotic membranes (Ilver et al. 1998; Mahdavi et al. 2002; Horridge et al. 2017; Yonezawa et al. 2017; Behrens et al. 2020). Such adhesive properties may contribute to stable association with host cells and structured microbial niches, although their specific contribution to *H. pylori* interactions with free‐living amoebae remains to be determined. Additional factors, including urease, LPS, γ‐glutamyl transpeptidase, and OMVs, collectively support immune evasion, oxidative stress resistance, and intercellular communication, traits consistent with persistence within protozoan hosts and structured microbial communities (Bury‐Moné et al. 2001; Mullaney et al. 2009; Cullen et al. 2011; Valenzuela et al. 2013; Yonezawa et al. 2017; Turner et al. 2018). Together, these factors provide mechanistic features that could support *H. pylori* persistence under diverse biological conditions, although direct evidence linking individual virulence factors to survival within free‐living amoebae remains limited (Figure 1).

**FIGURE 1:**
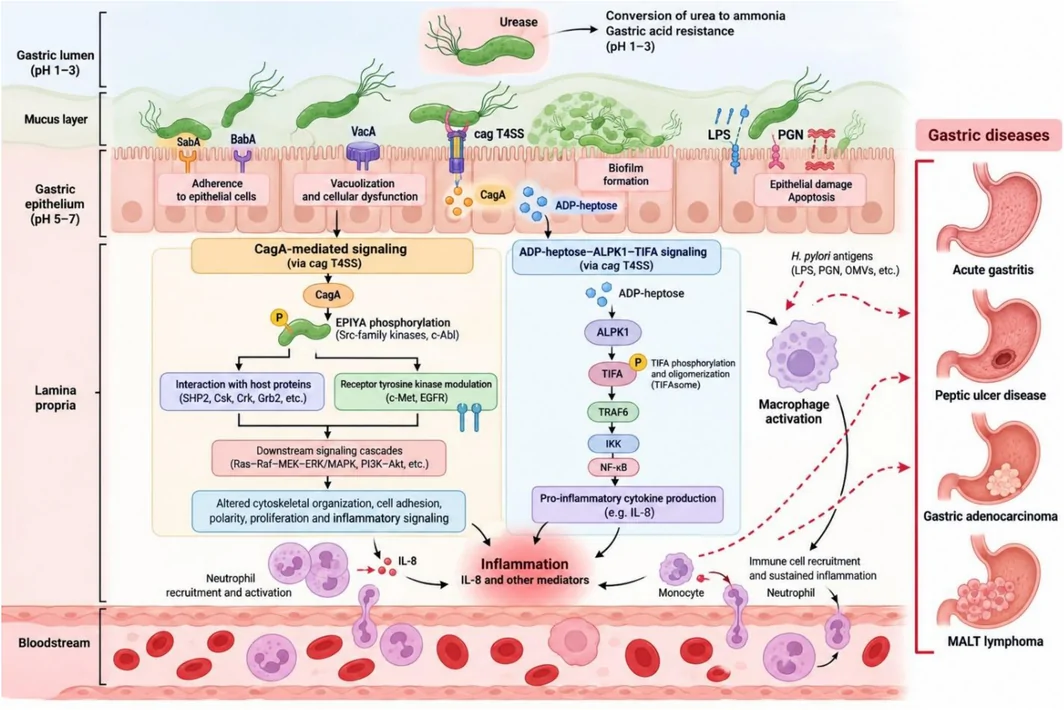
Schematic overview of the molecular and cellular events underlying *H. pylori* pathogenesis. In the gastric lumen and mucus layer, *H. pylori* survives acidic conditions (pH 1–3) primarily through urease activity, enabling colonisation of the gastric epithelium (pH 5–7). Adhesins such as BabA and SabA mediate bacterial adherence, while virulence factors, including VacA, induce epithelial vacuolisation. Translocation of CagA via the cag pathogenicity island‐encoded type IV secretion system (T4SS) triggers host signalling responses. Following translocation, CagA undergoes phosphorylation of its Glu‐Pro‐Ile‐Tyr‐Ala (EPIYA) motifs and perturbs receptor‐associated signalling and downstream kinase cascades, including the extracellular signal‐regulated kinase/mitogen‐activated protein kinase (ERK/MAPK) pathway. In parallel, the cag T4SS‐associated delivery of bacterial ADP‐heptose activates the alpha‐protein kinase 1 (ALPK1)‐TRAF‐interacting protein with forkhead‐associated domain (TIFA) axis, leading to TIFA signalling, nuclear factor kappa B (NF‐κB) activation, and pro‐inflammatory responses. These events promote interleukin‐8 (IL‐8) secretion, neutrophil chemotaxis, and macrophage activation within the lamina propria, amplifying gastric inflammation. *H. pylori* components such as lipopolysaccharide (LPS) and peptidoglycan (PGN), together with biofilm formation, contribute to epithelial damage, apoptosis, and immune dysregulation. Persistent inflammation and immune cell recruitment from the bloodstream drive disease progression, culminating in clinical outcomes such as acute gastritis, peptic ulcer disease, gastric adenocarcinoma, and mucosa‐associated lymphoid tissue (MALT) lymphoma. Created with BioRender.com.

### 2.3 Adaptive Survival Strategies: Biofilms and Intracellular Niches

*H. pylori* employs multiple adaptive strategies to withstand fluctuating environmental conditions, including acid resistance, biofilm formation, and intracellular survival. Acid adaptation is regulated through urease activity, the proton‐gated urea channel UreI, carbonic anhydrases, and stress‐response proteins that help maintain intracellular pH and protein homeostasis under acidic conditions (Bury‐Moné et al. 2001; Scott et al. 2002; Strugatsky et al. 2013). Biofilm formation creates structured microbial communities embedded within a protective extracellular matrix, enhancing *H. pylori* tolerance to environmental stresses and antimicrobial exposure and supporting the persistence of VBNC‐like cells capable of resuscitation (Figures 1 and 2) (Cole et al. 2004; Carron et al. 2006; Percival and Suleman 2014; Hathroubi et al. 2018). Although classically extracellular, *H. pylori* can invade epithelial cells and persist intracellularly, where it can interfere with lysosomal maturation and autophagy, thereby establishing protected intracellular niches (Odenbreit 2005; Terebiznik et al. 2009). This intracellular persistence may involve physiological adaptations that overlap with survival strategies used by bacteria that persist within free‐living amoebae. Consistent with this potential overlap, *H. pylori* has been shown experimentally to survive within *Acanthamoeba castellanii* and other free‐living amoebae, although the specific bacterial adaptations that enable persistence within amoebal hosts remain incompletely defined (Winiecka‐Krusnell et al. 2002; Moreno‐Mesonero et al. 2016; Dey et al. 2020).

**FIGURE 2:**
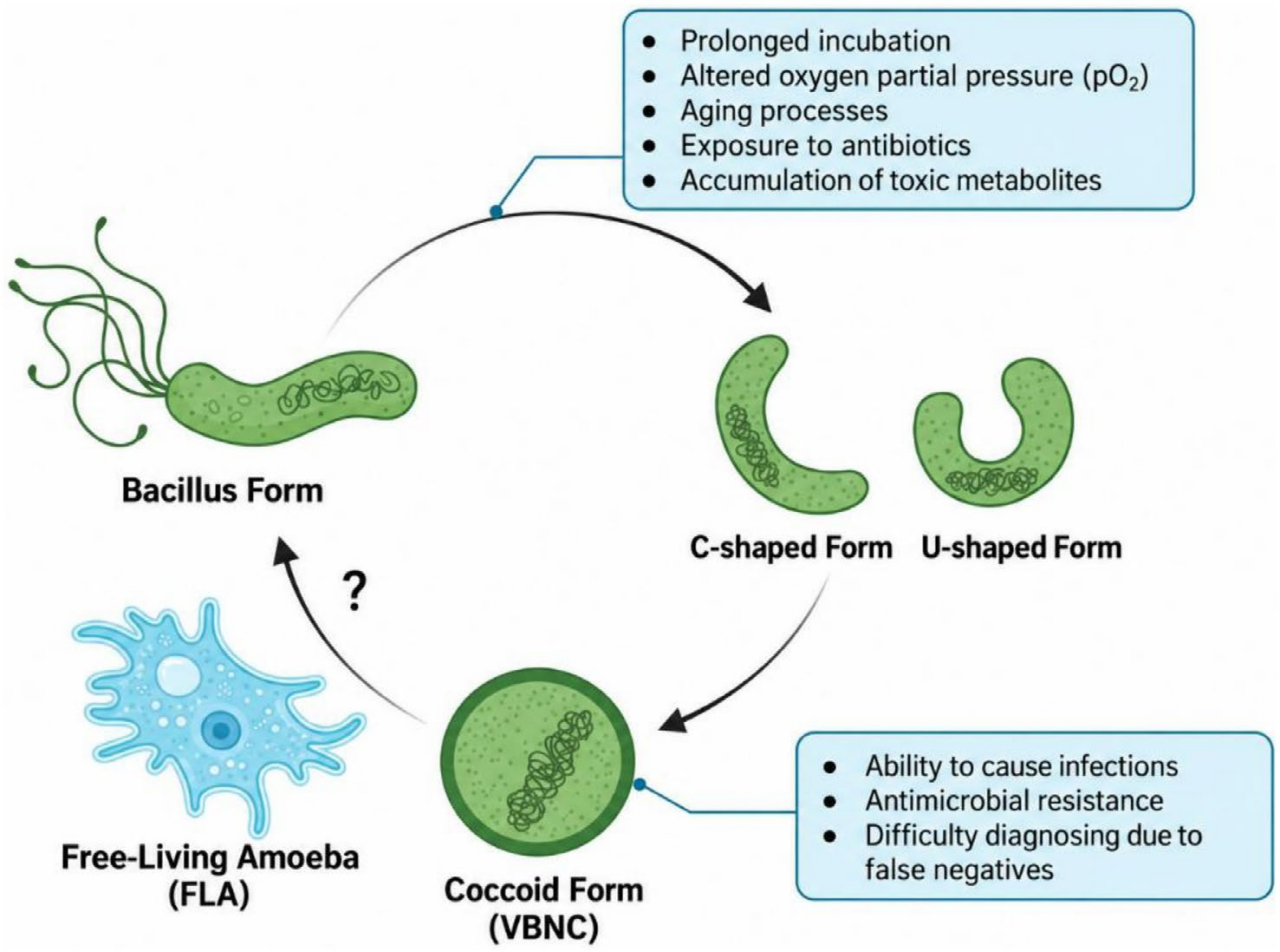
Dimorphism and morphological plasticity of *H. pylori* under environmental stress: Transition between spiral (S‐shaped) and coccoid (VBNC) forms. *H. pylori* predominantly exists in a spiral (S‐shaped) form under favourable conditions but can undergo progressive morphological changes, including C‐shaped and U‐shaped intermediates, ultimately converting into a coccoid form classified as viable but non‐culturable (VBNC). Adverse conditions such as prolonged incubation, altered oxygen partial pressure (pO_2_), where pO_2_ denotes the partial pressure of oxygen, aging, antibiotic exposure, and toxic metabolite accumulation promote this transition. The coccoid form is associated with enhanced stress tolerance, antibiotic resistance, persistence, and diagnostic challenges due to false‐negative culture results. The potential reversibility of the coccoid form back to the spiral (S‐shaped) morphology, including within host or environmental reservoirs such as free‐living amoebae (FLA), remains incompletely understood and is indicated by a question mark. Created with BioRender.com.

### 2.4 Coccoid and Viable but Non‐Culturable (VBNC) Forms of *H. pylori* : Dimorphism as a Strategy for Environmental Persistence

*H. pylori* exhibits pronounced morphological plasticity, existing primarily in two interconvertible forms: a spiral (S‐shaped), actively dividing form or a spherical coccoid form. This morphological dimorphism is associated with adaptation to changing environmental conditions (Figure 2). Similar to other bacterial pathogens such as *Salmonella*, *Campylobacter*, and *Escherichia coli* , *H. pylori* can enter a viable but non‐culturable (VBNC) state in response to adverse stimuli, including nutrient deprivation, oxidative stress, antibiotic exposure, and physicochemical stressors such as changes in oxygen availability (Ozçakir 2007; Li et al. 2014; Loke et al. 2016). As a microaerophilic bacterium, *H. pylori* is adapted to low‐oxygen conditions; deviations in oxygen partial pressure (pO_2_) from this preferred microaerobic environment can impose oxidative stress and favor morphological conversion toward coccoid/VBNC forms (Park et al. 2004).

During this transition, bacteria undergo coordinated alterations in cell size, morphology, respiration rate, nutrient uptake, gene expression, and biosynthetic activity, rendering them undetectable by conventional culture‐based methods while retaining metabolic viability (Fakruddin et al. 2013; Li et al. 2014; Loke et al. 2016). The coccoid form of *H. pylori* was first described in 1991 (Catrenich and Makin 1991) and is typically 0.5–1.4 μm in diameter (Willén et al. 2000). Subsequent ultrastructural and molecular studies confirmed the consistent presence of coccoid cells in both in vitro cultures and in vivo settings, although their functional significance remained controversial for several years (Sörberg et al. 1996; Kusters et al. 1997; Narikawa et al. 1997; Mizoguchi et al. 1999; Zheng et al. 1999). The transition from spiral (S‐shaped) to coccoid morphology is increasingly regarded as an active physiological response, rather than solely a passive degenerative process. *H. pylori* initiates coccoid conversion under moderately stressful conditions but fails to do so under extreme damage, supporting the view that coccoid formation represents a programmed survival response rather than cellular death (Bode et al. 1993; Azevedo et al. 2007; Faghri et al. 2014). Coccoid *H. pylori* cells retain key structural features of viable bacteria, including an intact cytoplasmic membrane, preserved cell wall, and organised cytoplasmic content, as demonstrated by transmission electron microscopy (Reshetnyak and Reshetnyak 2017). Their cytoplasm may contain electron‐dense inclusions enriched in polyphosphate, which can serve as intracellular energy reserves that protect essential macromolecules, including DNA, RNA, and proteins, during prolonged nutrient limitation (Willén et al. 2000; Loke et al. 2016). These adaptations highlight the metabolic and structural preparedness of coccoid forms for long‐term survival under non‐permissive conditions. Current models propose that *H. pylori* exists in at least three physiological states: viable culturable spiral cells, viable but non‐culturable coccoid cells, and degenerative, non‐viable forms (Cellini et al. 1994; Willén et al. 2000; Andersen and Rasmussen 2009; Loke et al. 2016). While early studies suggested that coccoid conversion represented irreversible cellular decline (Nilius et al. 1993; Kusters et al. 1997), subsequent work demonstrated that VBNC coccoid cells retain metabolic activity and, under favourable conditions, can resuscitate and regain infectivity (Cellini et al. 1994). Although aging coccoid forms may exhibit reduced urease activity and attenuated virulence (Nilius et al. 1993), this does not preclude their role in persistence and transmission. Importantly, coccoid and VBNC forms display enhanced resistance to antibiotic exposure and environmental stresses compared to actively dividing spiral cells, although the magnitude and biological consequences of this tolerance vary with the conditions used to induce the coccoid state (Krzyżek and Grande 2020; Sadeghloo et al. 2026). Despite reduced culturability, coccoid *H. pylori* maintains membrane potential, enzymatic activity, and expression of key virulence‐associated genes, including *cagA* and *vacA*, positioning these cells as metabolically poised for reactivation (Cellini et al. 1994; Azevedo et al. 2007; Sadeghloo et al. 2026).

From an ecological perspective, the coccoid/VBNC state may facilitate persistence of *H. pylori* outside the human host. These forms evade standard detection methods and survive in water systems, biofilms, and intracellular niches, where resuscitation may occur upon exposure to favourable conditions such as neutral pH or host‐derived signals (Cellini et al. 1994; Krzyżek and Grande 2020). Such traits overlap with survival strategies described for amoeba‐resistant bacteria within free‐living amoebae and provide a plausible physiological basis for persistence of coccoid *H. pylori* in FLA, as supported by experimental evidence of amoeba‐associated survival and recovery of non‐culturable cells (Moreno‐Mesonero et al. 2016; Dey et al. 2020).

Collectively, morphological plasticity and the VBNC state represent important adaptive strategies that may extend the ecological range of *H. pylori* , facilitating environmental survival and potentially contributing to transmission through environmental and protozoan reservoirs. Understanding the molecular regulation of coccoid formation, maintenance, and resuscitation is therefore critical for developing improved diagnostic approaches, antimicrobial strategies, and interventions to disrupt environmental persistence and reduce *H. pylori* transmission (Figure 3).

**FIGURE 3:**
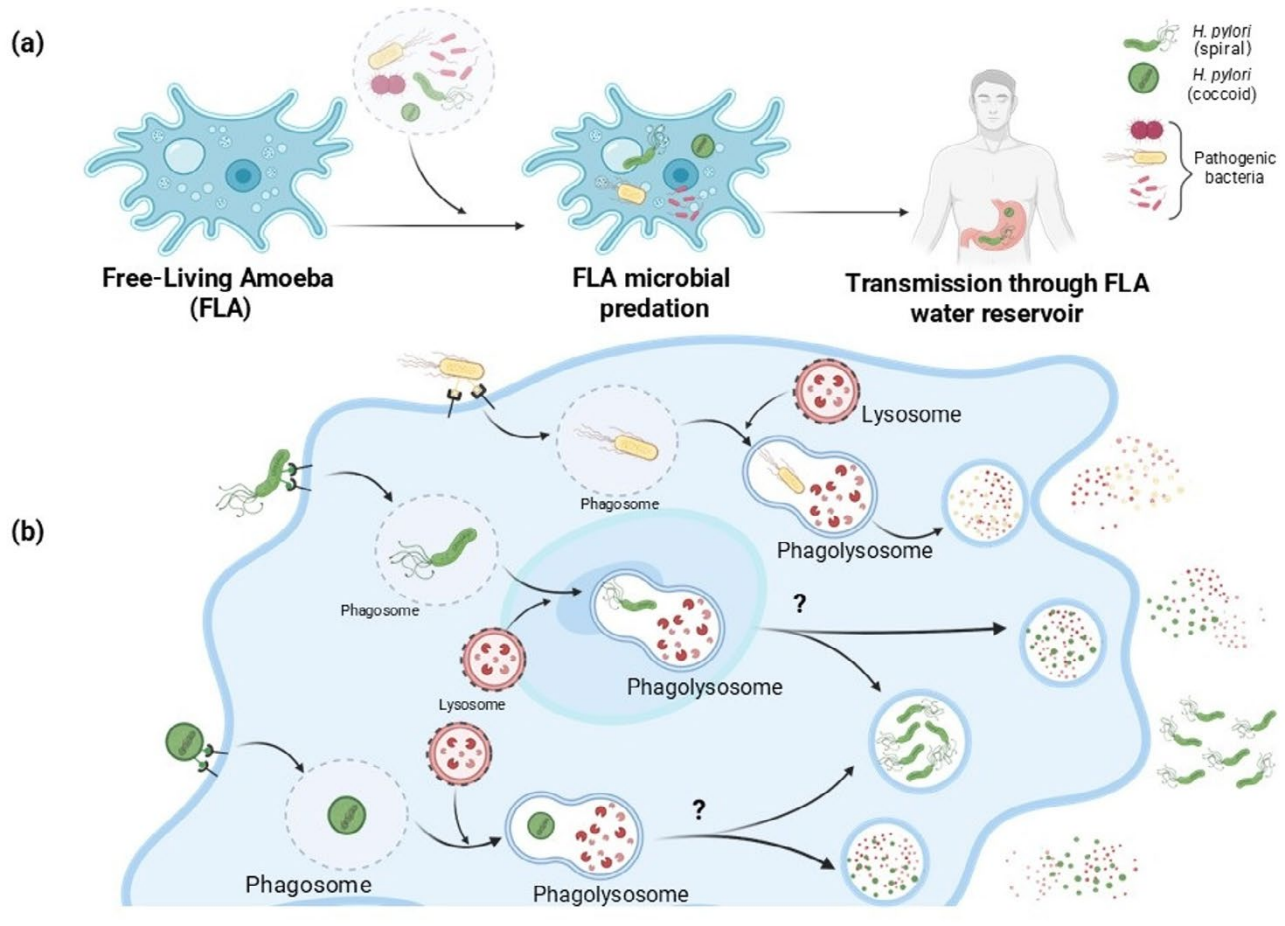
Schematic representation of free‐living amoeba‐bacteria interactions during phagocytosis, highlighting unresolved intracellular fates of *H. pylori* spiral (S‐shaped) and coccoid forms. (a) Free‐living amoebae (FLA) prey on diverse microbial communities in aquatic environments and can internalise pathogenic and non‐pathogenic bacteria, including *H. pylori* in both spiral (S‐shaped) and coccoid (viable but non‐culturable, VBNC) forms. Bacteria persisting within amoebae enable FLAs to act as environmental reservoirs and may facilitate human transmission through contaminated water. (b) Mechanistic overview of bacterial engulfment by FLA. Following uptake, bacteria are enclosed within phagosomes that may fuse with lysosomes to form phagolysosomes, resulting in the degradation of some pathogenic bacteria (yellow). The digested waste is finally released from the cell through exocytosis. The intracellular fate of *H. pylori* remains unclear. Question marks indicate unresolved mechanisms, including bacterial survival or replication within amoebal compartments and the potential reversion of coccoid to spiral (S‐shaped) forms, highlighting key knowledge gaps in *H. pylori* ‐FLA interactions. Created with BioRender.com.

## 3 Free‐Living Amoebae (FLA): Biology Relevant to *H. pylori* Ecology

FLA are a diverse group of unicellular, eukaryotic, heterotrophic microorganisms characterised by amoeboid motility mediated through pseudopodia (Loret and Greub 2010). They are ubiquitous in natural and anthropogenic environments, including freshwater, marine ecosystems, soil, wastewater, biofilms, and engineered water systems, where they contribute to microbial community structure through predation and other ecological interactions. While amoebae comprise a large and taxonomically diverse assemblage, only a subset is relevant to environmental microbiology and human health. Among environmentally relevant FLA, several important genera belong to the phylum Amoebozoa, including *Acanthamoeba* spp., *Vermamoeba vermiformis* , *Amoeba proteus* , and *D. discoideum* (Rodríguez‐Zaragoza 1994; Samba‐Louaka et al. 2019). Additional amoeboid taxa are found within the supergroup Excavata, including *Naegleria fowleri* and related genera such as *Vahlkampfia* and *Willaertia* (Adl et al. 2019). The ecological importance of FLA extends beyond bacterial predation. Their widespread distribution, persistence in biofilms, and ability to survive harsh environmental conditions enable them to function as environmental reservoirs for numerous amoeba‐resistant bacteria (ARB). These characteristics make FLA particularly relevant for understanding the environmental persistence and transmission of *H. pylori* (Moreno‐Mesonero et al. 2016; Dey et al. 2020). Molecular identification of FLA commonly relies on 18S rDNA sequencing for environmental detection and phylogenetic inference, complemented by additional loci when available (De Jonckheere and Brown 2005; Tyml et al. 2016; Denet et al. 2017).

### 3.1 Cellular Stages and Characteristics of Free‐Living Amoebae

Many environmentally relevant FLA alternate between an actively feeding trophozoite and a dormant cyst stage, although morphology and life‐cycle characteristics vary among taxa (Kang et al. 2017; Samba‐Louaka et al. 2019). The trophozoite is the metabolically active stage responsible for motility, bacterial recognition, phagocytosis, intracellular trafficking, and replication (Samba‐Louaka et al. 2019). Under unfavorable environmental conditions, including nutrient limitation, desiccation, osmotic stress, or temperature fluctuations, trophozoites can differentiate into resistant dormant cysts, enabling long‐term survival until favorable conditions return (Fouque et al. 2012) (Figure 4).

**FIGURE 4:**
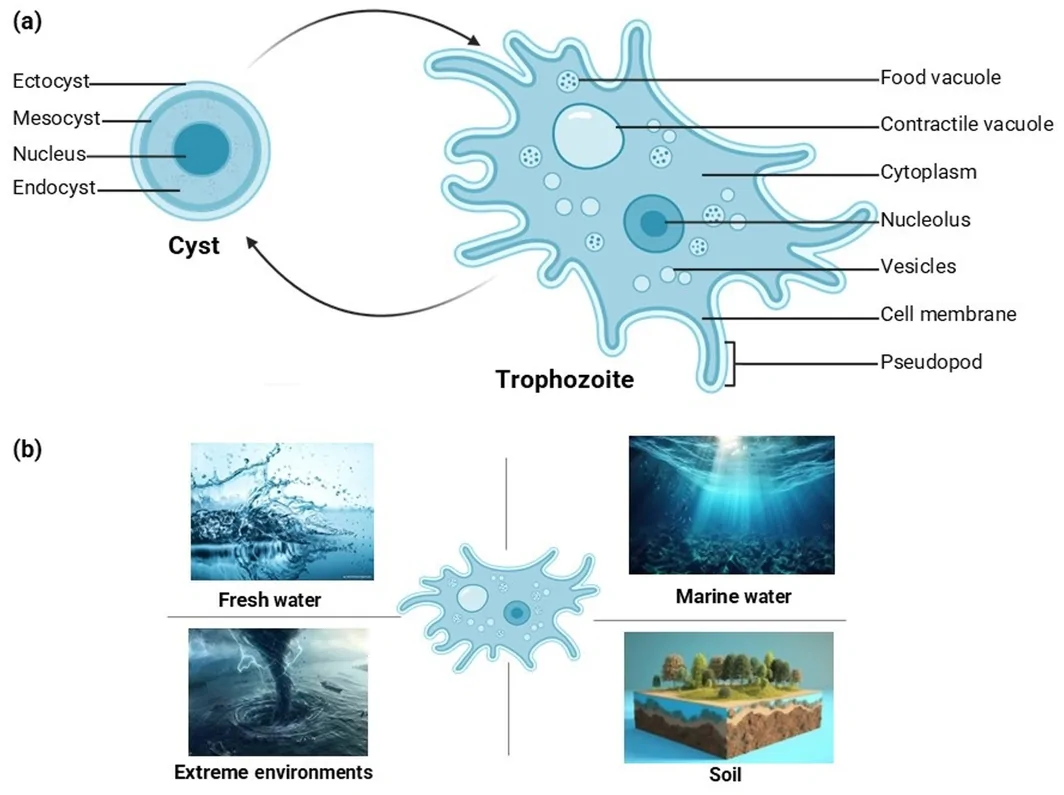
Free‐living amoeba (FLA) life cycle diagram. (a) The cyst structure is composed of three layers: endocyst, mesocyst, and ectocyst. The mesocyst varies by genus. The trophozoite is a metabolically active vegetative form. (b) Diagram of environments where FLA can be found. Created with BioRender.com.

Encystation represents an important ecological adaptation that enables FLA to withstand environmental stress and chemical disinfection (Dudley et al. 2005; Fouque et al. 2014). This differentiation process can be triggered by abiotic stressors and, in some amoebal systems, by biotic cues associated with microbial interactions or intracellular infection (El‐Etr et al. 2009; Lee et al. 2012). In *Acanthamoeba* spp., the cyst wall consists of a double‐layered structure composed of an ectocyst and an endocyst, enriched in cellulose and other polysaccharides, providing exceptional resistance to adverse environmental conditions (Chávez‐Munguía et al. 2005; Lemgruber et al. 2010). Encystation is accompanied by coordinated transcriptional and proteolytic remodelling, involving autophagy, cytoskeletal reorganisation, and synthesis of cyst‐specific components (Fouque et al. 2012; Lloyd 2014; Bernard et al. 2022). Upon restoration of favourable conditions, excystation regenerates metabolically active trophozoites (Mattar and Byers 1971; Fouque et al. 2015). Importantly, cysts can act as environmental shelters for intracellular bacteria, protecting them from desiccation, disinfectants, and antimicrobial agents. Several bacterial pathogens, including *Legionella pneumophila* , *Mycobacterium* spp., *Vibrio cholerae* , and *Francisella tularensis* , have been shown to persist within amoebal cysts, facilitating long‐term environmental survival and potential re‐emergence (Kilvington and Price 1990; Abd et al. 2005; El‐Etr et al. 2009; Medie et al. 2011; Hennebique et al. 2021). In contrast, current evidence suggests that *H. pylori* survival is primarily associated with trophozoites, whereas evidence for persistence within mature amoebal cysts remains limited. Thus, the contribution of encystment to *H. pylori* persistence remains insufficiently understood (Moreno‐Mesonero et al. 2016; Dey et al. 2020).

### 3.2 Amoebal Predation and Amoeba‐Resistant Bacteria

FLA are professional phagocytes that feed on a wide range of microorganisms, including bacteria, fungi, algae, and other protists, in both planktonic and biofilm‐associated states (Huws et al. 2005; Sanders et al. 2017; Shi et al. 2021). Their prey spectrum encompasses diverse bacterial phyla, and predation efficiency varies with prey characteristics and environmental context (Weekers et al. 1993; Brock et al. 2018). Prey are recognised through chemotaxis and surface interactions before being engulfed into membrane‐bound phagosomes that normally mature into degradative phagolysosomes (Akya et al. 2009; Kuburich et al. 2016; Vines and King 2019; Vij et al. 2020). However, amoebae exhibit considerable plasticity in predatory strategies, adjusting their responses according to prey identity and environmental conditions (Lamrabet et al. 2020; Jauslin et al. 2021).

Despite efficient predation, numerous microorganisms have evolved mechanisms to resist amoebic digestion. These organisms, collectively termed amoeba‐resistant bacteria (ARB), can exploit amoebae as protective environmental reservoirs and, in some cases, replicate intracellularly (Ly and Müller 1990; Birtles et al. 2000; Horn et al. 2000; Landers et al. 2000; Greub and Raoult 2004; Huws et al. 2006) (Figure 5). Representative ARB include *L. pneumophila* , *Mycobacterium* spp., *Vibrio cholerae* , *Francisella tularensis* , and *Pseudomonas aeruginosa* (Cateau et al. 2014) (Table 1). Because several aspects of phagosomal trafficking are conserved between amoebae and mammalian phagocytic cells, adaptation to intracellular survival within FLA is thought to facilitate subsequent survival in mammalian phagocytic cells. Consequently, amoebae have been proposed as environmental ‘training grounds’ for intracellular pathogens. More broadly, experimental studies indicate that exposure to amoebal predation can select for bacterial traits associated with stress resistance and altered physiology, suggesting that amoebal interactions may shape bacterial adaptation beyond intracellular survival (Inglis et al. 2000; Bunma et al. 2023). These studies demonstrate that FLA serve not only as microbial predators but also as environmental reservoirs that enhance bacterial persistence, facilitate adaptation to environmental stress, and potentially promote horizontal gene transfer among co‐inhabiting bacteria (Horn et al. 1999, 2001; Collingro et al. 2005; Delafont et al. 2015; Mehari et al. 2016; Schulz et al. 2016; Tsao et al. 2017; Rahman et al. 2022). Beyond these interactions, environmental *Acanthamoeba* populations can harbour diverse bacterial endosymbionts belonging to several phylogenetically distinct lineages, highlighting the broad diversity of amoeba‐bacterium associations in natural environments (Amann et al. 1997; Schmitz‐Esser et al. 2008).

**FIGURE 5:**
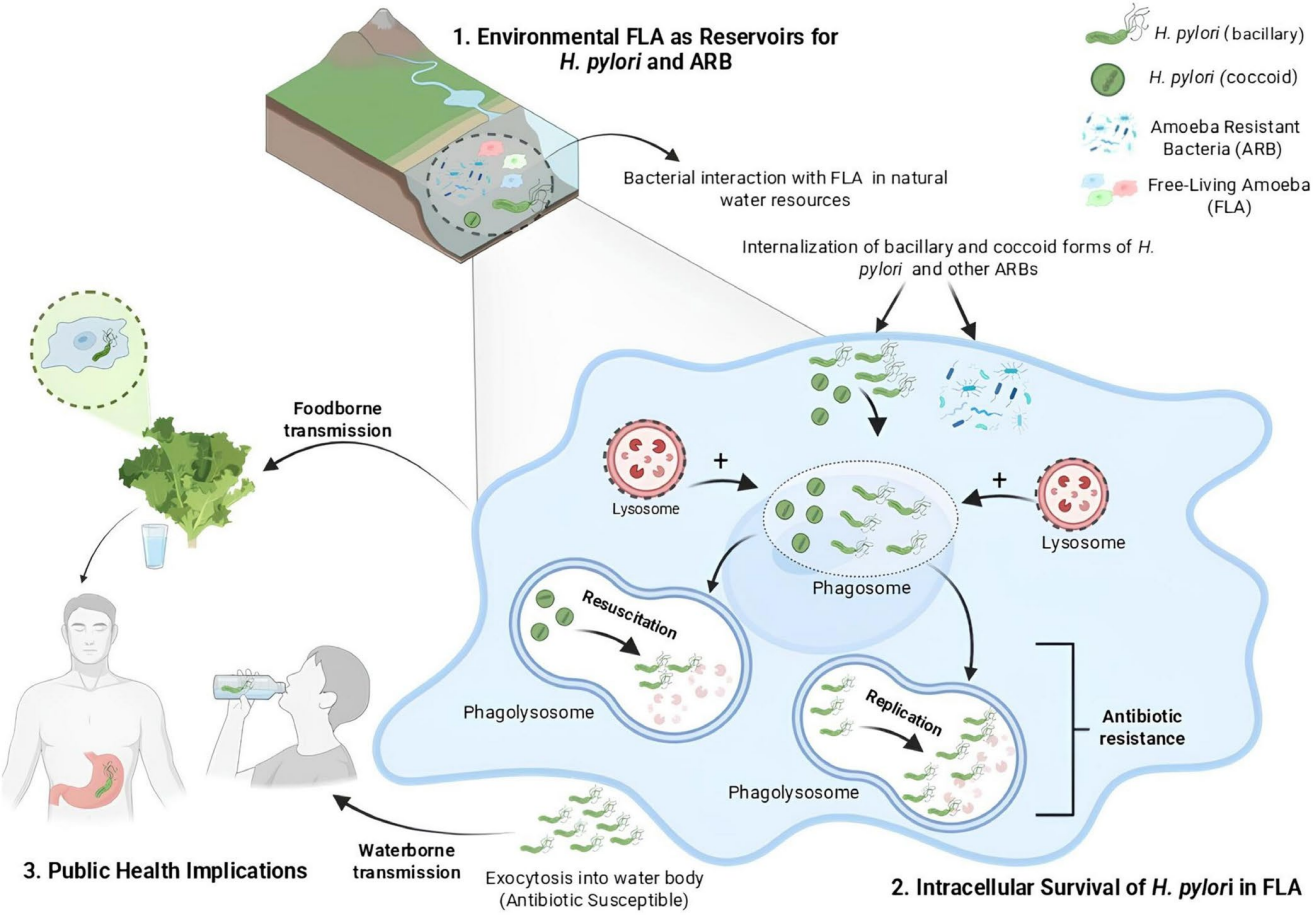
Schematic overview of the proposed role of free‐living amoebae (FLA) in the environmental persistence and transmission of *H. pylori* and its public health relevance. In aquatic environments, both spiral (S‐shaped) and coccoid (viable but non‐culturable, VBNC) forms of *H. pylori* may be internalised by FLA during microbial predation, thereby potentially protecting them from environmental stresses, disinfectants, and antibiotics. Following phagocytosis, bacteria are enclosed within intracellular compartments, including phagosomes and phagolysosomes. While some bacteria may be degraded, *H. pylori* is proposed to survive and possibly replicate within amoebal cells, with unresolved questions regarding intracellular persistence and resuscitation from the coccoid to the spiral form. Release of bacteria from amoebae into water systems or onto food surfaces may facilitate waterborne and foodborne transmission, highlighting FLA as an environmental reservoir that could contribute to *H. pylori* persistence and dissemination. Created with BioRender.com.

**TABLE 1:** List of bacteria resistant to amoebal predation.

Amoeba‐resistant bacteria	Amoebal host genera	Amoeba–bacteria relationship	Pathogenicity to humans	References
Actinobacteria				
*Mycobacterium*	*Acanthamoeba*, *D. discoideum*	Parasitism	Established	(Drancourt 2014; Thomas and McDonnell 2007)
*Rhodococcus*	*Pelomyxa*	Unknown	Unknown	(Gutiérrez et al. 2017)
Alphaproteobacteria				
*Afipia felis*	*Acanthamoeba*	Parasitism	Potential	(La Scola and Raoult 1999)
*Afipia broomeae*	*Acanthamoeba*	Parasitism	Potential	(Scola et al. 2002)
*Caedibacter caryophilus*	*Acanthamoeba*	Endosymbiosis	Unknown	(Horn et al. 1999)
*Jidaibacter*	*Acanthamoeba*	Endosymbiosis	Unknown	(Schulz et al. 2016)
*Nucleicultrix*	*Acanthamoeba*	Endosymbiosis	Unknown	(Schulz et al. 2014)
*Odyssella*	*Acanthamoeba*	Endosymbiosis	Unknown	(Birtles et al. 2000)
*Paracaedibacter*	*Acanthamoeba*	Endosymbiosis	Unknown	(Horn et al. 2001)
Bacteroidetes				
*Amoebophilus*	*Acanthamoeba*	Parasitism	Unknown	(Horn et al. 2001)
*Flavobacterium*	*Acanthamoeba*	Endosymbiosis	Unknown	(Horn et al. 2001)
Betaproteobacteria				
*Burkholderia cepacia*	*Acanthamoeba*	Parasitism	Established	(Landers et al. 2000)
*Burkholderia pseudomallei*	*Acanthamoeba*	Parasitism	Established	(Bunma et al. 2023; Inglis et al. 2000)
*Procabacter*	*Acanthamoeba*	Endosymbiosis	Unknown	(Schmitz‐Esser et al. 2008)
*Ralstonia pickettii*	*Acanthamoeba*	Parasitism	Established	(Michel and Hauröder 1997)
Chlamydia				
*Neochlamydia*	*Acanthamoeba*, *V. vermiformis*	Endosymbiosis/Parasitism	Potential	(Horn et al. 2000)
*Mesochlamydia*	*Vannella*	Parasitism	Unknown	(Corsaro et al. 2013)
*Parachlamydia*	*Acanthamoeba*	Endosymbiosis	Potential	(Amann et al. 1997)
*Protochlamydia*	*Acanthamoeba*, *Naegleria*	Endosymbiosis	Potential	(Collingro et al. 2005)
Deltaproteobacteria				
*Helicobacter pylori*	*Acanthamoeba and other genera*	Endosymbiosis	Unknown	(Moreno‐Mesonero et al. 2017; Winiecka‐Krusnell et al. 2002)
Dependentiae				
*Vermiphilus pyriformis*	*V. vermiformis*	Endosymbiosis	Unknown	(Delafont et al. 2015)
Firmicutes				
*Listeria monocytogenes*	*Acanthamoeba*	Parasitism	Established	(Ly and Müller 1990)
*Staphylococcus aureus*	*Acanthamoeba*	Parasitism	Established	(de Souza et al. 2017; Huws et al. 2006)
Gammaproteobacteria				
*Acinetobacter baumannii*	*Acanthamoeba*	Parasitism	Established	(Cateau et al. 2011)
*Berkheya*	*Acanthamoeba*	Endosymbiosis	Unknown	(Mehari et al. 2016)
*Coxiella burnetii*	*Acanthamoeba*	Parasitism	Established	(Tsao et al. 2017)
*Escherichia coli* O157	*Acanthamoeba*	Parasitism	Established	(Barker et al. 1999)
*Francisella*	*Acanthamoeba*	Parasitism	Established	(Hennebique et al. 2021)
*Legionella anisa*	*Acanthamoeba*	Parasitism	Established	(La Scola et al. 2001)
*Legionella pneumophila*	*Acanthamoeba*, *D. discoideum*, *V. vermiformis* , *Naegleria*	Parasitism	Established	(Best and Abu Kwaik 2018)
*Stenotrophomonas maltophilia*	*Acanthamoeba*, *Naegleria*, *Vermamoeba*, *Willaertia*	Parasitism	Established	(Corsaro et al. 2013; Denet et al. 2018)
*Vibrio cholerae*	*Acanthamoeba*, *Naegleria*	Parasitism	Established	(Thom et al. 1992; Van der Henst et al. 2016)

Among ARB, *L. pneumophila* represents the best‐characterised model, replicating within multiple amoebal genera before lysing the host and disseminating into aquatic environments (Rowbotham 1980; La Scola et al. 2001; Best and Abu Kwaik 2018; Swart et al. 2018). Similar intracellular interactions have also been described for *Mycobacterium* spp., *Vibrio cholerae* , *Acinetobacter baumannii* , and other bacterial pathogens (Thom et al. 1992; Cateau et al. 2011; Corsaro et al. 2013; Drancourt 2014; Van der Henst et al. 2016; de Souza et al. 2017; Denet et al. 2018). Amoebae not only provide a replicative environment but can also enhance bacterial persistence by protecting pathogens within resistant cysts (King et al. 1988; Lambrecht et al. 2015). Moreover, intra‐amoebal cohabitation of multiple bacterial species may create opportunities for HGT, potentially accelerating the dissemination of antibiotic resistance genes (Saisongkorh et al. 2010; Rahman et al. 2022). The widespread occurrence of FLA in natural and engineered environments, together with their ability to protect intracellular bacteria, underscores their ecological importance as environmental reservoirs of microbial pathogens with potential implications for environmental persistence and transmission (La Scola and Raoult 1999; Thomas et al. 2010; Cateau et al. 2014; Muchesa et al. 2017). However, most current evidence is derived from laboratory co‐culture studies using a limited number of amoebal species, and further investigations under environmentally relevant conditions are needed to fully understand the ecological significance of FLA‐bacteria interactions. Collectively, these characteristics establish FLA as important environmental reservoirs for intracellular bacteria and provide the biological framework for understanding interactions between FLA and *H. pylori* , discussed in the following sections.

## 4 *H. pylori* and Amoeba Interaction

### 4.1 Amoebal Internalisation of *H. pylori* and Protection From Environmental Stress

Emerging microbiological and ecological studies provide growing evidence for cellular and environmental interactions between *H. pylori* and FLA (Table 2). Collectively, these findings suggest that FLA may serve as protective environmental reservoirs capable of supporting the viability of both spiral (culturable) and coccoid (viable but non‐culturable, VBNC) forms of *H. pylori* . Amoebal internalisation can enhance bacterial survival under adverse environmental conditions, including exposure to disinfectants, and may potentially facilitate morphological switching and persistence in aquatic and food‐associated environments (Figure 5). Early experimental work demonstrated that *H. pylori* can survive and proliferate within *A. castellanii* under non‐ideal culture conditions, leading to the hypothesis that amoebae provide a sheltered niche that protects the bacterium from environmental stress (Winiecka‐Krusnell et al. 2002). Subsequent studies confirmed that *H. pylori* can be phagocytosed by other amoebal species, including *A. polyphaga* , although intracellular persistence was strain‐ and host‐species‐dependent, highlighting biological variability in these interactions (Smith and Ashbolt 2012). Further strengthening the reservoir hypothesis, Moreno‐Mesonero et al. demonstrated that amoebal internalisation confers protection from standard chlorination treatment, suggesting that FLA may act as a protected waterborne reservoir for *H. pylori* (Moreno‐Mesonero et al. 2016). More recent co‐culture experiments further showed enhanced bacterial survival and maintenance of the spiral or helical morphology of *H. pylori* during association with *A. castellanii* , with loss of the survival advantage upon physical separation of the two organisms, indicating that direct interaction with amoebae supports bacterial fitness and is accompanied by differential *H. pylori* gene expression during co‐culture (Hojo et al. 2020).

**TABLE 2:** List of cellular and environmental interactions between *H. pylori* and FLA.

Amoeba species	*H. pylori* strain/form	Main findings	Implication	References
*Acanthamoeba castellanii*	67:21	Survives and propagates within amoebae under stress conditions.	Amoebae act as protective environmental reservoirs.	(Winiecka‐Krusnell et al. 2002)
*Acanthamoeba polyphaga*	ATCC 43504	Phagocytosed but limited intracellular survival; species‐dependent interaction.	Interaction varies by amoeba species; limited persistence observed.	(Smith and Ashbolt 2012)
*Acanthamoeba castellanii* (ATCC 30010)	NCTC 11638	Internal survival and chlorination resistance within amoebae.	Supports hypothesis of amoebae as waterborne reservoirs.	(Moreno‐Mesonero et al. 2016)
Free‐living amoebae (environmental mix)	Environmental isolates	Viable *H. pylori* detected in > 50% of FLA‐positive water samples.	Demonstrates the role of amoebae as vectors in real environmental settings.	(Moreno‐Mesonero et al. 2017)
*Acanthamoeba castellanii* (Neff strain)	TK1402 & ATCC 43504 (spiral and coccoid forms)	Enhanced survival and coccoid stabilisation in co‐culture with *A. castellanii*.	Direct contact promotes morphological stability and gene regulation.	(Hojo et al. 2020)
*Willaertia magna* and *Vermamoeba vermiformis*	G27 & mutants (*cag* Pathogenicity Island (*cag*PAI) genes)	Replication and VBNC resuscitation in acidified phagosomes; vesicle‐mediated release.	Confirms intracellular replication and persistence of coccoid forms in amoebae.	(Dey et al. 2020)

### 4.2 Morphological Switching and Intracellular Replication of *H. pylori*

Beyond passive protection, interaction with amoebae may actively influence *H. pylori* viability, morphology, and replication (Figure 2). Mechanistic studies demonstrated that *H. pylori* can replicate within acidified phagosomes of specific amoebal hosts, including *Willaertia magna* and *Vermamoeba vermiformis* (Dey et al. 2020). Notably, this intracellular environment supports the resuscitation of coccoid VBNC forms, which are otherwise associated with environmental dormancy and reduced metabolic activity (Dey et al. 2020). These findings suggest that amoebal phagosomes can provide conditions conducive to both persistence and reactivation. Following intracellular replication, *H. pylori* was observed to be released from amoebae within extracellular vesicles, a mechanism that may enhance environmental dissemination while providing additional protection against external stressors (Dey et al. 2020). Although these observations are currently limited to a small number of experimental systems, they provide important proof‐of‐concept evidence that amoebae can act not only as passive shelters but also as active intracellular niches supporting *H. pylori* survival and morphological plasticity.

### 4.3 Convergence With Established Amoeba‐Resistant Bacteria: *Legionella* and *Mycobacterium*

The ability of *H. pylori* to survive and replicate within FLA suggests parallels with the broader conceptual framework of ARBs, a group that includes well‐established amoeba‐resistant bacteria such as *L. pneumophila* and environmental *Mycobacterium* species (Swart et al. 2018; Dey et al. 2020). In these organisms, amoebae serve as intracellular training grounds that can select for traits that enhance resistance to phagosomal stress and, in some cases, virulence in mammalian hosts. The interaction mechanisms of these established ARBs therefore offer a useful parallel to *H. pylori* 's emerging intracellular survival strategy. *L. pneumophila* , the causative agent of Legionnaires' disease, is a natural intracellular parasite of FLA. It employs a highly specialised Dot/Icm Type IV Secretion System to inject effector proteins that actively remodel the nascent phagosome into the permissive Legionella‐Containing Vacuole (LCV) (Swart et al. 2018; Lockwood et al. 2022). This process allows intracellular replication and survival, and the amoeba host acts as an evolutionary ‘training ground’ that enhances the bacterium's virulence and survival in human macrophages (Price et al. 2024). Similarly, members of the genus *Mycobacterium*, including *M. avium* , also exploit FLA, resisting phagosomal degradation and, alongside *L. pneumophila* , exploiting host lipid droplets as an intracellular nutrient source to establish a replicative niche (Bosch et al. 2021).

While *L. pneumophila* and several *Mycobacterium* species represent highly evolved, obligate or facultative intracellular pathogens, *H. pylori's* newly demonstrated ability to replicate within phagosomes, together with evidence that the Type IV secretion system component CagE contributes to growth within amoebae, suggests potential mechanistic convergence in the exploitation of the amoebal niche for environmental survival and transmission (Dey et al. 2020). Notably, FLA‐mediated protection from biocidal stress is observed across diverse pathogens, including *H. pylori* , *Legionella* spp., and *Mycobacterium* spp. (Moreno‐Mesonero et al. 2016; Swart et al. 2018). These parallels highlight the ecological importance of FLA as protective intracellular niches and suggest that amoebal association may represent a general strategy for bacterial persistence under environmental stress.

### 4.4 Epidemiological Implications and Transmission

Environmental surveys support the potential public health relevance of *H. pylori* ‐FLA interactions. Viable *H. pylori* has been detected in more than 50% of FLA‐positive water samples, including samples derived from treated drinking water systems (Moreno‐Mesonero et al. 2017). Beyond water, amoebal‐associated *H. pylori* has also been identified on fresh produce such as lettuce, suggesting that FLA may act as potential carriers of *H. pylori* in both water‐ and food‐associated environments (Moreno‐Mesonero et al. 2020). Together, these observations support a model in which FLA provide a protective refuge for *H. pylori* , enabling survival during environmental exposure, resistance to disinfection, morphological switching, and potential re‐establishment of infectivity when favourable conditions arise.

## 5 Implications of Interaction Between *H. pylori* and FLA

The demonstrated association between *H. pylori* and FLA carries significant implications for bacterial ecology, epidemiology, and public health management.

### 5.1 Persistence in Harsh Environments

The intracellular association with amoebae can provide protection that may extend *H. pylori's* persistence outside the human host (Figure 5). The sequestration within the amoebal cytoplasm, and potentially within the protective cyst stage, shields the bacterium from high concentrations of chemical disinfectants, such as chlorine, used in water treatment facilities (Moreno‐Mesonero et al. 2016). Moreover, this intracellular niche likely buffers against other environmental stressors, including temperature fluctuations, pH changes, exposure to solar UV radiation, and antibiotics in environmental settings; although direct evidence for protection against these stressors remains limited. The resuscitation of VBNC coccoid forms within amoebal phagosomes further highlights the potential role of FLA in overcoming dormancy and maintaining long‐term viability in aquatic systems (Dey et al. 2020).

### 5.2 Transmission and Reservoirs

The detection of viable *H. pylori* in FLA‐positive water samples, including treated drinking water, strongly supports the potential role of waterborne amoebae as environmental reservoirs for the gastric pathogen (Figure 5) (Moreno‐Mesonero et al. 2017). The detection of amoebal‐associated *H. pylori* on lettuce also supports a potential role for FLA in the environmental persistence and dissemination of *H. pylori* in food‐associated settings (Moreno‐Mesonero et al. 2020). Evidence from other amoeba‐associated bacterial pathogens further demonstrates the potential public‐health relevance of these interactions, with patients in intensive‐care settings shown to be exposed to FLA‐associated pathogens (Scola et al. 2002). The release of *H. pylori* via extracellular vesicles further provides another potential mechanism for bacterial dispersal in aquatic environments and may offer additional protection from external stressors (Dey et al. 2020).

### 5.3 Impact on Virulence

The physical interaction and intracellular residence of *H. pylori* within amoebae may have consequences beyond passive protection. Co‐culture experiments indicate that this association supports bacterial survival and alters gene expression (Hojo et al. 2020). Given that many ARBs, such as *L. pneumophila* , acquire enhanced virulence traits (e.g., resistance to human macrophages) through their amoebal interaction, it is hypothesised that *H. pylori* may similarly undergo changes in gene expression while residing within the amoeba. These changes could potentially involve upregulating factors that mediate resistance to the acidic gastric environment, enhancing adhesion to human epithelial cells, or modulating immune responses, thereby increasing the survival fitness of the released *H. pylori* progeny. The finding that a *cagE* mutant, lacking a functional component of the pathogenicity‐associated Type IV secretion system, showed little growth in amoebae further supports a potential link between known virulence factors and fitness within the amoebal niche (Dey et al. 2020).

## 6 Concluding Remarks

Although free‐living amoebae (FLA) have been the subject of extensive investigation, research on these microorganisms has often focused on their pathogenic potential and their ability to host and support the replication and survival of various human bacterial pathogens (Greub and Raoult 2004; Cateau et al. 2014). However, their ubiquitous distribution in natural and engineered environments, together with their ecological role as microbial predators, underscores their broader significance in environmental microbiology. Beyond regulating microbial community structure through predation (Rodríguez‐Zaragoza 1994; Samba‐Louaka et al. 2019; Shi et al. 2021), FLA can also function as environmental reservoirs that promote bacterial persistence, adaptation, and evolution, although the broader ecological consequences of these interactions remain comparatively understudied (Weekers et al. 1993; Rosenberg et al. 2009; Brock et al. 2018).

Evidence accumulated over the past two decades demonstrates that FLA exhibit remarkable diversity in their interactions with bacteria. While several studies have shown that environmental amoebae can efficiently prey upon and eliminate bacterial populations, including antibiotic‐resistant clinical isolates (Bornier et al. 2021), other bacteria have evolved strategies to evade digestion and establish intracellular persistence within amoebal hosts (Greub and Raoult 2004; Cateau et al. 2014). These contrasting interactions highlight that FLA are not universally permissive hosts but dynamic microbial predators whose responses vary according to amoebal species, bacterial characteristics, and environmental conditions (Lamrabet et al. 2020; Jauslin et al. 2021). Consequently, exercise caution when extrapolating findings from laboratory co‐culture systems employing axenic amoebal strains, as these models may not fully reflect the ecological complexity of naturally occurring FLA populations (Bloomfield et al. 2015).

Within this broader ecological context, increasing evidence indicates that *H. pylori* can survive within selected FLA species, supporting the concept that amoebae may serve as potential environmental reservoirs that facilitate bacterial persistence under adverse conditions. However, the molecular mechanisms governing intracellular survival, the contribution of amoebal encystment to long‐term bacterial protection, the pathways involved in vesicular trafficking and bacterial release, and the potential resuscitation of viable but non‐culturable (VBNC) *H. pylori* remain incompletely understood. Addressing these questions will be essential for clarifying the environmental persistence of *H. pylori* and the potential contribution of FLA to its transmission in aquatic and engineered water systems.

Future investigations should prioritise environmentally relevant FLA species and incorporate advanced imaging, transcriptomics, proteomics, and metagenomic approaches to characterise the molecular dialogue between *H. pylori* and its amoebal hosts. In parallel, standardised FLA‐ *H. pylori* co‐culture models should be developed to systematically compare different amoebal species and environmental conditions and to assess bacterial persistence, intracellular replication, VBNC resuscitation, and potential transmission. Studies integrating biofilm models, environmental surveillance, and complex microbial communities will provide a more realistic understanding of FLA‐ *H. pylori* interactions than conventional laboratory co‐culture systems. Such efforts will not only advance our understanding of microbial persistence and adaptation but also contribute to improved environmental monitoring, microbial risk assessment, and water safety management strategies. Collectively, elucidating the ecological relationship between FLA and *H. pylori* will strengthen our understanding of environmental microbial ecology and provide new insights into the persistence and potential environmental transmission of this globally important pathogen.

## Author Contributions

**Otmane Lamrabet:** conceptualization, visualization, methodology, investigation, formal analysis, data curation, writing – original draft, writing – review and editing, funding acquisition. **Meriem Bekliz:** visualization, formal analysis, data curation, writing – review and editing. **Shamsul Qumar:** data curation, writing – review and editing, formal analysis, methodology. **Merylin Sebastian:** visualization, writing – review and editing. **Ula Abdurahman:** visualization, writing – review and editing. **Mohamad Hamad:** visualization, formal analysis, data curation, writing – review and editing. **Lucy Semerjian:** investigation, data curation, writing – review and editing. **Ghalia Khoder:** conceptualization, investigation, visualization, methodology, formal analysis, data curation, writing – original draft, writing – review and editing, funding acquisition.

## Funding

This work was supported by University of Sharjah (25011102121, 25021450170).

## Conflicts of Interest

The authors declare no conflicts of interest.

## Data Availability

The data analysed in this review are obtained from publicly available sources and are appropriately cited in the manuscript.
